# Supplementary material for: The French version of the Gilles de la Tourette Syndrome Quality of Life Scale for adolescents (GTS-QOL-French-Ado): Adaptation and psychometric evaluation
Source: PLoS One. 2022 Nov 30;17(11):e0278383. doi: 10.1371/journal.pone.0278383 (PMC9710837; doi:10.1371/journal.pone.0278383)
Supplement: S1 File — (PDF) [file pone.0278383.s001.pdf]

The GTS-QOL-French-Ado questionnaire

Les problèmes de santé peuvent affecter la qualité de vie d'une personne de différentes manières. Ce questionnaire étudie la manière dont votre maladie influe sur votre bien-être.
Merci de mettre une croix dans la case correspondant à la réponse la plus adaptée à votre ressenti et de répondre à **toutes les questions**.
Il est à noter que cette liste inclut de nombreux problèmes que vous ne rencontrerez probablement jamais.

Durant les quatre dernières semaines avez-vous :	Aucun problème	Problème léger	Problème modéré	Problème important	Problème très important
1. été incapable de contrôler tous vos mouvements ?	☐	☐	☐	☐	☐
2. eu des difficultés dans vos activités de la vie quotidienne ou vos loisirs (comme cuisiner, écrire) ?	☐	☐	☐	☐	☐
3. souffert de douleurs ou de blessures physiques dus à vos tics ?	☐	☐	☐	☐	☐
4. été gêné(e) par des bruits que vous ne pouvez-vous empêcher de faire ?	☐	☐	☐	☐	☐
5. redouté de dire des gros mots que vous n'aviez pas l'intention de dire ?	☐	☐	☐	☐	☐
6. redouté de faire quelque chose d'embarrassant (comme des gestes grossiers)	☐	☐	☐	☐	☐
7. été obligé(e) de répéter des mots encore et encore ?	☐	☐	☐	☐	☐
8. été obligé(e) de refaire ou de répéter des choses que d'autres personnes font ou disent (comme copier, imiter des gens) ?	☐	☐	☐	☐	☐
9. été obligé(e) de faire des choses d'une manière répétée et d'une certaine façon (par exemple vérifier plusieurs fois) ?	☐	☐	☐	☐	☐
10. eu des pensées ou des images déplaisantes vous passant par la tête ?	☐	☐	☐	☐	☐
11. eu des difficultés de concentration ?	☐	☐	☐	☐	☐
12. eu des problèmes de mémoire ?	☐	☐	☐	☐	☐
13. perdu ou égaré des objets importants (comme un portefeuille, des clefs, un téléphone mobile) ?	☐	☐	☐	☐	☐
14. eu des difficultés à finir ce que vous avez commencé?	☐	☐	☐	☐	☐
15. eu l'impression dans l'ensemble d'être en mauvaise santé ?	☐	☐	☐	☐	☐

Durant les quatre dernières semaines avez-vous :	Aucun problème	Problème léger	Problème modéré	Problème important	Problème très important
16. été triste ou déprimé ?	☐	☐	☐	☐	☐
17. ressenti des changements d'humeur rapides ?	☐	☐	☐	☐	☐
18. ressenti un manque de confiance en soi ?	☐	☐	☐	☐	☐
19. Vous êtes-vous senti(e) angoissé(e) ?	☐	☐	☐	☐	☐
20. Vous êtes-vous senti(e) énervé(e) ?	☐	☐	☐	☐	☐
21. eu des difficultés à garder votre calme?	☐	☐	☐	☐	☐
22. eu l'impression de ne pas contrôler votre vie ?	☐	☐	☐	☐	☐
23. Vous êtes-vous senti(e) frustré(e) (contrarié(e)) ?	☐	☐	☐	☐	☐
24. ressenti le besoin de plus d'aide ou de soutien des autres?	☐	☐	☐	☐	☐
25. eu des difficultés à voir vos amis ?	☐	☐	☐	☐	☐
26. eu des difficultés à prendre part à des activités sociales (comme aller manger à l'extérieur) ?	☐	☐	☐	☐	☐
27. eu le sentiment d'être seul(e) ou isolé(e) ?	☐	☐	☐	☐	☐

Merci d'indiquer à quel point vous vous sentez dans l'ensemble satisfait de votre vie en ce moment, en mettant une croix entre 0 et 100 sur la ligne ci-dessous.

0 10 20 30 40 50 60 70 80 90 100

☹ ☺

Extrêmement insatisfait de ma vie — Extrêmement satisfait de ma vie

Avez-vous rempli ce questionnaire?

☐ **seul(e)**

☐ **Avec l'aide de quelqu'un, précisez :..**
