# Supplementary figures and images for: The French version of the Gilles de la Tourette Syndrome Quality of Life Scale for adolescents (GTS-QOL-French-Ado): Adaptation and psychometric evaluation
Source: PLoS One. 2022 Nov 30;17(11):e0278383. doi: 10.1371/journal.pone.0278383 (PMC9710837; doi:10.1371/journal.pone.0278383)

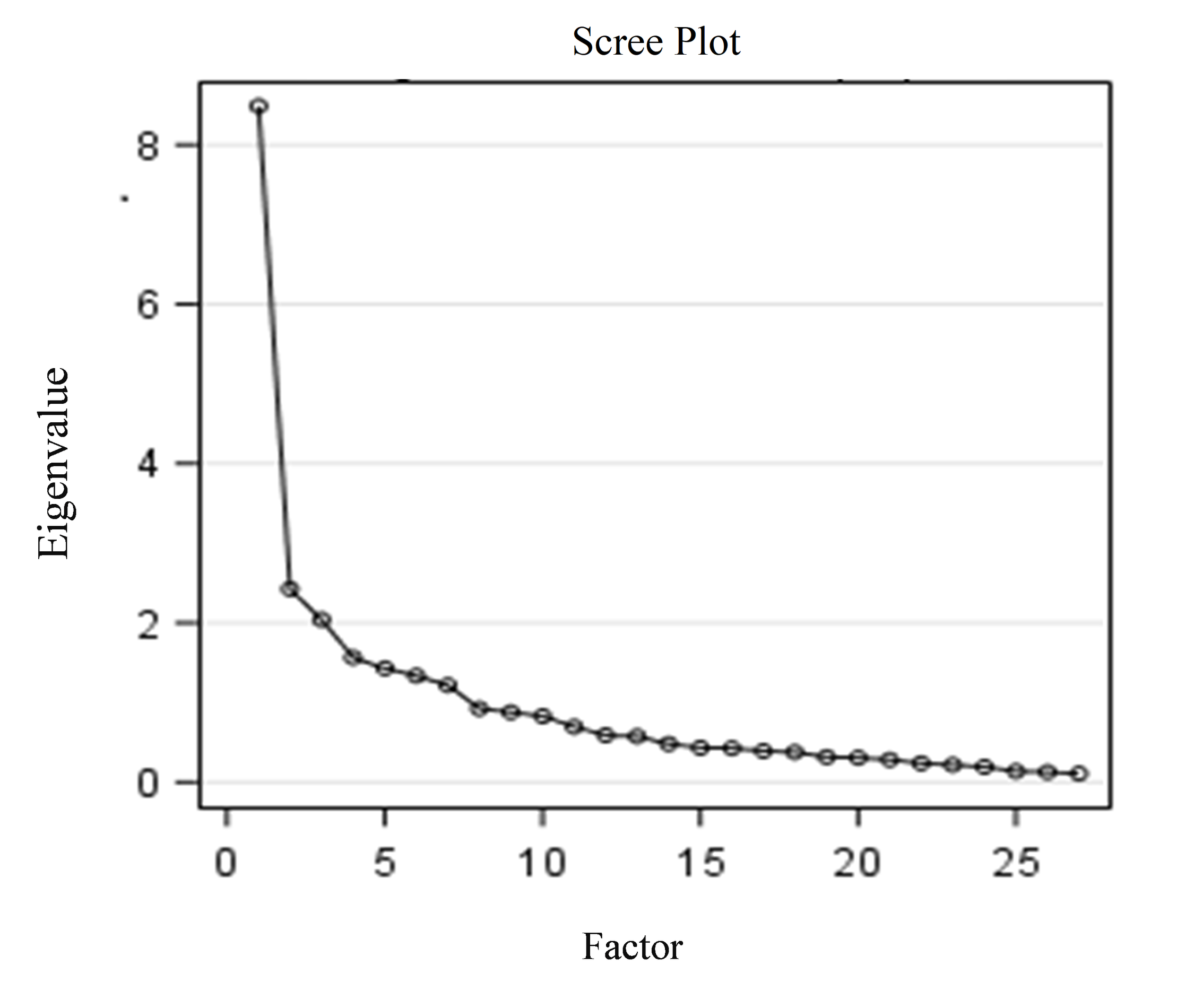

Supplement: S1 Fig — (TIF) [file pone.0278383.s002.tif]
