# Supplementary material for: The French version of the Gilles de la Tourette Syndrome Quality of Life Scale for adolescents (GTS-QOL-French-Ado): Adaptation and psychometric evaluation
Source: PLoS One. 2022 Nov 30;17(11):e0278383. doi: 10.1371/journal.pone.0278383 (PMC9710837; doi:10.1371/journal.pone.0278383)
Supplement: S1 Table — (DOCX) [file pone.0278383.s003.docx]

**S1 Table. Factor loadings from the factor analysis of the GTS-QOL-French-Ado items**

|  | | Factor 1 | Factor 2 | Factor 3 | Factor 4 | Factor 5 |
| --- | --- | --- | --- | --- | --- | --- |
|  | Variance explained (%) | 31.4 | 9.0 | 7.5 | 5.8 | 5.3 |
| **‘Psychological’ subscale** | |  |  |  |  |  |
| 9. Repeating actions | | **0.40** | -0.10 | -0.20 | 0.27 | **0.39** |
| 10. Unpleasant thoughts | | **0.63** | 0.05 | 0.17 | -0.11 | 0.16 |
| 15. Concerns about poor health | | **0.41** | **0.41** | -0.12 | -0.05 | -0.20 |
| 17. Mood switches | | **0.71** | 0.08 | -0.02 | 0.15 | -0.08 |
| 19. Anxiety | | **0.40** | 0.30 | 0.23 | -0.09 | 0.18 |
| 20. Restlessness | | **0.84** | -0.01 | -0.05 | 0.07 | 0.03 |
| 21. Temper dyscontrol | | **0.79** | -0.14 | 0.06 | 0.13 | 0.14 |
| 22. Lack of control over own life | | **0.57** | 0.29 | 0.21 | 0.10 | -0.19 |
| 23. Frustration | | **0.50** | 0.27 | 0.25 | 0.10 | -0.07 |
| **‘Social’ subscale** | |  |  |  |  |  |
| 16. Depressed mood | | **0.36** | **0.57** | 0.02 | -0.10 | 0.12 |
| 18. Lack of self-confidence | | 0.23 | **0.49** | -0.04 | 0.12 | 0.15 |
| 24. Lack of social support | | 0.08 | **0.54** | 0.02 | 0.31 | 0.22 |
| 25. Difficulty seeing friends | | -0.18 | **0.86** | 0.07 | 0.05 | 0.01 |
| 26. Difficulty taking part in social activities | | -0.01 | **0.82** | 0.14 | 0.03 | -0.02 |
| 27. Loneliness/isolation | | 0.12 | **0.68** | -0.02 | -0.04 | 0.15 |
| **‘Echo-coprophenomena/Obsessive-compulsive’ subscale** | |  |  |  |  |  |
| 4. Phonic tics | | -0.26 | 0.14 | **0.63** | 0.06 | 0.25 |
| 5. Involuntary swearing | | 0.14 | -0.04 | **0.86** | -0.11 | -0.10 |
| 6. Embarrassing gestures | | 0.04 | 0.22 | **0.73** | -0.18 | 0.07 |
| 7. Repeating words | | 0.17 | -0.10 | **0.61** | 0.26 | 0.04 |
| 8. Copying people | | 0.03 | -0.03 | **0.66** | **0.34** | 0.01 |
| **‘Cognitive’ subscale** | |  |  |  |  |  |
| 11. Difficulty concentrating | | 0.16 | 0.23 | 0.01 | **0.32** | **0.39** |
| 12. Memory problems | | 0.07 | 0.02 | -0.05 | **0.70** | 0.16 |
| 13. Losing important things | | 0.09 | -0.14 | 0.25 | **0.66** | -0.15 |
| 14. Difficulty finishing tasks | | 0.01 | 0.27 | -0.03 | **0.68** | -0.03 |
| **‘Physical’ subscale** | |  |  |  |  |  |
| 1. Movement dyscontrol | | 0.15 | -0.19 | 0.26 | -0.28 | **0.72** |
| 2. Difficulty in daily life activities | | 0.04 | 0.14 | 0.01 | 0.11 | **0.59** |
| 3. Pain or injuries | | -0.11 | 0.17 | -0.01 | 0.03 | **0.64** |

Loadings equal to or higher than 0.32 are presented in bold.
