# Supplementary material for: The French version of the Gilles de la Tourette Syndrome Quality of Life Scale for adolescents (GTS-QOL-French-Ado): Adaptation and psychometric evaluation
Source: PLoS One. 2022 Nov 30;17(11):e0278383. doi: 10.1371/journal.pone.0278383 (PMC9710837; doi:10.1371/journal.pone.0278383)
Supplement: S2 Table — (DOCX) [file pone.0278383.s004.docx]

**S2 Table. Corrected item-total correlations for the GTS-QOL-French-Ado subscales**

| Subscales | Item | Psychological | Social | Echo-coprophenomena / Obsessive-compulsive | Cognitive | Physical |
| --- | --- | --- | --- | --- | --- | --- |
| Psychological | 9 | **0.37** | 0.28 | 0.20 | 0.37 | 0.23 |
|  | 10 | **0.62** | 0.41 | 0.35 | 0.30 | 0.28 |
|  | 15 | **0.43** | 0.42 | -0.01 | 0.18 | 0.10 |
|  | 17 | **0.65** | 0.39 | 0.22 | 0.44 | 0.15 |
|  | 19 | **0.54** | 0.58 | 0.42 | 0.35 | 0.34 |
|  | 20 | **0.74** | 0.48 | 0.25 | 0.43 | 0.21 |
|  | 21 | **0.70** | 0.38 | 0.31 | 0.45 | 0.31 |
|  | 22 | **0.69** | 0.58 | 0.35 | 0.43 | 0.21 |
|  | 23 | **0.65** | 0.56 | 0.46 | 0.42 | 0.27 |
| Social | 16 | 058 | **0.67** | 0.32 | 0.36 | 0.34 |
|  | 18 | 0.55 | **0.57** | 0.27 | 0.47 | 0.28 |
|  | 24 | 0.49 | **0.62** | 0.29 | 0.56 | 0.39 |
|  | 25 | 0.34 | **0.65** | 0.29 | 0.30 | 0.27 |
|  | 26 | 0.43 | **0.74** | 0.32 | 0.39 | 0.25 |
|  | 27 | 0.47 | **0.65** | 0.31 | 0.33 | 0.35 |
| Echo-coprophenomena / Obsessive-compulsive | 4 | 0.18 | 0.29 | **0.50** | 0.23 | 0.40 |
|  | 5 | 0.32 | 0.24 | **0.69** | 0.18 | 0.21 |
|  | 6 | 0.35 | 0.33 | **0.65** | 0.24 | 0.33 |
|  | 7 | 0.36 | 0.27 | **0.54** | 0.29 | 0.32 |
|  | 8 | 0.29 | 0.22 | **0.58** | 0.32 | 0.20 |
| Cognitive | 11 | 0.49 | 0.46 | 0.26 | **0.42** | 0.41 |
|  | 12 | 0.38 | 0.44 | 0.18 | **0.58** | 0.20 |
|  | 13 | 0.25 | 0.19 | 0.30 | **0.39** | 0.09 |
|  | 14 | 0.43 | 0.50 | 0.13 | **0.48** | 0.16 |
| Physical | 1 | 0.22 | 0.20 | 0.41 | 0.12 | **0.41** |
|  | 2 | 0.31 | 0.39 | 0.41 | 0.32 | **0.31** |
|  | 3 | 0.16 | 0.32 | 0.20 | 0.21 | **0.39** |

Correlations of items with their parent subscale (corrected for overlap) are in bold.
