# Supplementary material for: The French version of the Gilles de la Tourette Syndrome Quality of Life Scale for adolescents (GTS-QOL-French-Ado): Adaptation and psychometric evaluation
Source: PLoS One. 2022 Nov 30;17(11):e0278383. doi: 10.1371/journal.pone.0278383 (PMC9710837; doi:10.1371/journal.pone.0278383)
Supplement: Attachment — Submitted filename: Response to Reviewers 2.docx [file pone.0278383.s007.docx]

Reviewer #1: Thank you for the opportunity to review the revised version of the manuscript titled ‘French version of the Gilles the la Tourette Syndrome Quality of Life Scale for adolescents’.

This version represents a significant improvement where all my previous comments have been addressed.

However, I do have a new minor comment/suggestion. Specifically, in the statistical analysis section, the authors state "statistical analyses were […] conducted at a two-sided alpha 0.05 sig level”. It would good be clarify which analyses is this statement referring to.

I wish the authors good luck with their future publications.

We thank the reviewer whose suggestions helped to improve the article.

We have specified the analysis to which the significance level referred to in the statistical analysis section: “*Statistical analyses were performed with SAS v9.4 software (SAS Institute) and conducted at a two-sided alpha = 0.05 significance level to test the null hypothesis of zero correlation between two variables*”.
