# Supplementary material for: The French version of the Gilles de la Tourette Syndrome Quality of Life Scale for adolescents (GTS-QOL-French-Ado): Adaptation and psychometric evaluation
Source: PLoS One. 2022 Nov 30;17(11):e0278383. doi: 10.1371/journal.pone.0278383 (PMC9710837; doi:10.1371/journal.pone.0278383)
Supplement: Attachment — Submitted filename: Response to the Editor.docx [file pone.0278383.s008.docx]

**Journal Requirements**

*Please review your reference list to ensure that it is complete and correct. If you have cited papers that have been retracted, please include the rationale for doing so in the manuscript text, or remove these references and replace them with relevant current references. Any changes to the reference list should be mentioned in the rebuttal letter that accompanies your revised manuscript. If you need to cite a retracted article, indicate the article’s retracted status in the References list and also include a citation and full reference for the retraction notice.*

**Response:** All the references have been checked: no cited papers have been retracted. Two changes have been done in the reference list: the journal names of the references 17 and 21 have been modified.

**To the editor**

***Comment 1:*** *Please report the 95%CI of ICC in the abstract.*

**Response 1:** As recommended, we reported in the abstract the 95% CI of ICC.

***Comment 2:*** *In the conclusion of the abstract, please be a little more specific with the sentence: “Further psychometric testing is recommended to confirm our first results”. Indeed, it is surprising to read this sentence just after giving results of psychometric testing in your own study. Which psychometric properties should be tested again? Or why this should be tested again? Please details.*

**Response 2:** We modified the sentence to highlight that further studies on responsiveness to change would be of great interest.

***Comment 3:*** *In page 4, phase 1, please adapt the sentence “Our team cross-culturally adapted the GTS-QOL in adults (GTS-QOL-French)” with “Our team translated in French and cross-culturally adapted the GTS-QOL in adults (GTS-QOL-French)”. It will make the sentence more clear.*

**Response 3:** We modified the sentence as suggested.

***Comment 4****: It is not clear what was the time interval used for test-retest reliability. In the method section, authors mentioned 2 weeks. However, in the results section, authors mentioned 1 month.*

**Response 4:** For test-retest reliability, the GTS-QOL-French-Ado questionnaire was mailed a second time 15 days after the first assessment. Then the adolescents returned by mail their completed questionnaire. We considered for test-retest reliability analysis only the questionnaires received by mail within 15 days, which corresponds to one month after the first completion. We agree that this was not clear and we modified the sentence in the results section: “*For the reliability assessment, the retest was sent to 46 participants. Of the 36 (78.3%) who returned completed questionnaires within 15 days, four reported that their health status had degraded between test and retest*”.

***Comment 5:*** *Moreover, it is not clear why only a sample of respondents were invited to complete a second time the questionnaire. The sample size for test-restest reliability is therefore too small to obtain valid results.*

**Response 5:** We first invited a sample of respondents to complete a second time the GTS-QOL-French-Ado questionnaire. The response rate was very low and we decided in consequence to invite all adolescents subsequently included to complete a second time the questionnaire. Unfortunately, the resulted number of participants in the test-retest reliability was lower than the one attempted, that is 50 participants.

However, as highlighted in the discussion section, in the adapted COSMIN Risk of Bias checklist ^1^, no recommendation was made on the sample size required for reliability analysis. Despite the small sample size for the reliability analysis, good agreement was obtained for all the subscales.

^1^ Mokkink LB, de Vet HCW, Prinsen CAC, Patrick DL, Alonso J, Bouter LM, Terwee CB. COSMIN Risk of Bias checklist for systematic reviews of Patient-Reported Outcome Measures. Qual. Life Res. 2018; 27:1171–1179.

***Comment 6:*** *In the results section, please merge the two paragraph about (i.e. the paragraph presenting the number of participants included in this analysis and the paragraph presenting results of reliability).*

**Response 6**: A reviewer asked us previously to report concisely in one section all the participants’ data. As suggested here, we merged the paragraph presenting the number of participants included in the reliability analysis and the paragraph presenting the results of reliability under Reliability subheading in Results section.

***Comment 7:*** *Please report the percentage of hypotheses confirmed for convergent validity. It is suggested that a convergent validity is ensured if at least 75% of hypotheses are met.*

**Response 7**: As suggested, we reported the percentage of hypotheses confirmed for convergent validity under Convergent validity subheading in Results section. We also added in the Discussion section that 92.2% of hypotheses were met.

***Comment 8:*** *Are the French versions of the CDI and MASC validated ? I asked the question because authors provided results of psychometric analyses of the French version of the MOVES but for the two other scales used.*

**Response 8:** The French versions of the CDI and MASC have been validated by Mack et al. ^1^ and Turgeon et al. ^2^ respectively. We agree with the editor that it was not clear as we reported results of psychometric analysis of the French version of the MOVES. We deleted this sentence.

^1^ Mack K, Moor L. Versions françaises d’échelles d’évaluation de la dépression: Les échelles CDI et ISC de Maria Kovacs. 1982;30:627‑652.

^2^ Turgeon L, Chartrand É, Robaey P, Gauthier AK. [Psychometric properties of a French version of the Multidimensional Anxiety Scale for Children (MASC).]. Revue Francophone de Clinique Comportementale et Cognitive. 2006;11.

***Comment 9:*** *Regarding sociodemographic data, I absolutely do not understand the sentence “All data are available at Mendeley [29]”.*

**Response 9:** We provided here the public repository where the data underlying the findings presented in the manuscript are held. It is a data availability statement describing where the data can be found. If it is not clear, this sentence could be deleted, as the data availability statement could be found elsewhere in PLoS ONE publication.

***Comment 10:*** *Regarding factorial analysis, it is not clear if previous work already proposed a theoretical model for the original scale. If yes, a CFA should be performed instead of a EFA (see one previous comment of reviewer for which authors did not replied “Terwee et al. (2007) also suggest that where a theoretical model exists, confirmatory factor analysis should be used. As this study modelled the adaptation of a pre-existing scale for adults to an adolescent population, and given the authors confidence in the results of the EFA, use of CFA could be justified and would provide further credit to the design of the scale”. I have the same comment, I do not understand why authors did not performed a CFA since a theoretical model already exists”.*

**Response 10**: We did not think that a theoretical model already exists as factor structures of the original GTS-QOL and the French version of the GTS-QOL for adults were different. The same applies for adolescents with two different factor structures of the English and Italian versions of the C&A-GTS-QOL. In the framework of a CFA, a hypothesized model is established and the CFA confirm that it provides a good fit to the observed data. This makes CFA a method suited for validation of instruments with a predefined measurement model. Or, given previous studies on the GTS-QOL for adults or adapted for adolescents that found different factor structures, such hypothesized model seems lacking to us. Moreover, the perception and definition of HRQoL may vary from adults to adolescents and could impact the factorial structure of the scale. So we preferred to conduct an EFA to explore the possible underlying factor structure of a set of observed variables without imposing a preconceived structure on the outcome.

Another reason to not have performed a CFA is the sample size of our study, which is a major inconvenience to conduct a CFA. Anthoine et al. ^1^ reported that recommendations for the sample size when conducting a CFA are disparate, ranging from 150 to 1000 subjects. The large sample size is a more crucial requirement in CFA compared to EFA ^2^.

^1^ Anthoine E, Moret L, Regnault A, Sbille V, Hardouin JB. Sample size used to validate a scale: a review of publications on newly-developed patient reported outcomes measures. Health and Quality of Life Outcomes. 2014;12:176.

^2^ Kyriazos TA. Applied Psychometrics: Sample Size and Sample Power Considerations in Factor Analysis (EFA, CFA) and SEM in General. Psychology. 2018; 9:2207-2230.
