# Supplementary material for: The French version of the Gilles de la Tourette Syndrome Quality of Life Scale for adolescents (GTS-QOL-French-Ado): Adaptation and psychometric evaluation
Source: PLoS One. 2022 Nov 30;17(11):e0278383. doi: 10.1371/journal.pone.0278383 (PMC9710837; doi:10.1371/journal.pone.0278383)
Supplement: Attachment — Submitted filename: Response to the Editor.docx [file pone.0278383.s009.docx]

**To the editor**

*Journal Requirements:*

*Please review your reference list to ensure that it is complete and correct. If you have cited papers that have been retracted, please include the rationale for doing so in the manuscript text, or remove these references and replace them with relevant current references. Any changes to the reference list should be mentioned in the rebuttal letter that accompanies your revised manuscript. If you need to cite a retracted article, indicate the article’s retracted status in the References list and also include a citation and full reference for the retraction notice.*

**Response:** Two references have been added (numbered 51 and 52).

*I would be able to accept the publication of the manuscript once the authors will have reported more precisely in the manuscript their response to my comments n° 5 and 10. It is appreciated to provide me explicit answers but this should be reported transparently in the manuscript as well.*

**Response:** We have reported in the manuscript our responses to the previous comments n°5 (about the sample size of respondents for test-retest reliability) and n°10 (about our choice to not perform a confirmatory factor analysis).

Responses to comment n°5 were reported under Reliability subheading in the Results section and in the Discussion section.

Responses to comment n°10 were reported in the Discussion section.
